# Ultrasensitive fluorescent aptasensor for CRP detection based on the RNase H assisted DNA recycling signal amplification strategy

**DOI:** 10.1039/c9ra01352k

**Published:** 2019-04-16

**Authors:** Zhongzhi Liu, Dan Luo, Fangling Ren, Fengying Ran, Wei Chen, Bingqiang Zhang, Ceming Wang, Hao Chen, Jian Wei, Qinhua Chen

**Affiliations:** Affiliated Dongfeng Hospital, Hubei University of Medicine Hubei Shiyan 442008 China cqh77@163.com +86 0719 8272283; College of Pharmacy, Hubei University of Medicine Hubei Shiyan 442008 China; Hubei Key Laboratory of Wudang Local Chinese Medicine Research, Hubei University of Medicine Hubei Shiyan 442008 China

## Abstract

An aptamer-based method for the ultrasensitive fluorescence detection of C-reactive protein (CRP) was developed using the ribonuclease H (RNase H) assisted DNA recycling signal amplification strategy. In this assay, CRP can specifically bind to the aptamer of CRP and the DNA chain of P1 is released from the aptamer/P1 (Ap/P1) complexes. After the addition of the fluorescence labeled (5-FAM) RNA, P1 hybridizes with fluorescence labeled RNA to form a P1/RNA double strand. When RNase H is added, the RNA with fluorescence labeling in the double strand is specifically cut into nucleotide fragments, which cannot be adsorbed on the surface of the GO, so as to generate a fluorescence signal. In the absence of CRP, fluorescence labeled RNA cannot hybridize with P1 to form double strands, which is able to directly adsorb on the surface of GO, resulting in no fluorescence signal. The detection limit is as low as 0.01 ng mL^−1^, with a linear dynamic range from 50 pg mL^−1^ to 100 ng mL^−1^. This sensor is able to detect CRP in spiked human serum, urine and saliva. Thus, it shows a great application prospect in disease diagnosis and prognosis.

## Abstract 2

An aptamer-based method for the ultrasensitive fluorescence detection of C-reactive protein (CRP) was developed using the ribonuclease H (RNase H) assisted DNA recycling signal amplification strategy.

## Graphical abstract

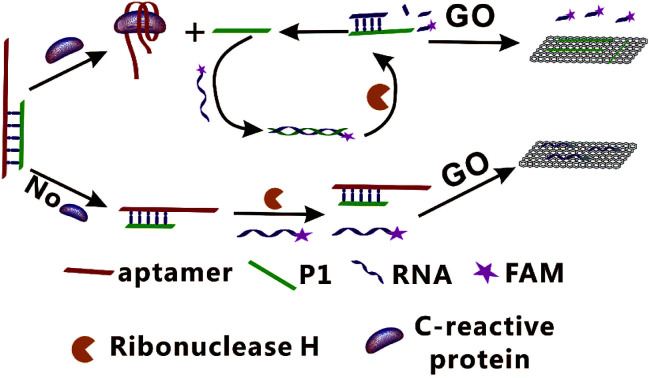

## 1 Introduction

Rapid and reliable detection of protein biomarkers in biofluid samples plays a vital role in diagnosis and management of disease. Sensitive detection and quantification of C-reactive protein (CRP) in an easy, cheap, and fast way is highly desired in clinical diagnostics in order to prevent serious inflammatory states.^1^

CRP is a general inflammatory biomarker, and is a major acute-phase reactant protein produced in the liver and a recent study has shown that the blood concentration of CRP slightly increases in patients with atherosclerosis.^2^ The most widely used CRP diagnostic methods are antibody-based assays.^2^ At present, several techniques including the use of a capacitive biosensor,^3^ microfluidic biosensor,^4–6^ electrochemical biosensor,^7–9^ sandwich immunoassay,^10,11^ fluorescent biosensor,^12^ chemiluminescence immunoassay,^13^ micro-fluxgate sensor,^14^ fiber optic biosensor,^15^ magnetoimpedance-based biosensor,^16^ nanowire-mesh biosensor^17^ and electrochemical impedance spectroscopy^18^ have been developed for the detection of CRP. However, these methods for CRP detection are usually time-consuming, labor-intensive, complex in operation or preparation and involve expensive reagents.^3–8^ Aptamer-based analytical methods for a broad range of targets have attracted increasing attention since the aptamer was first reported in 1990, and have been applied in bio-analyses, diagnoses, therapies, drug development, and environmental and food analyses.^2^ However, these methods still have disadvantages such as low sensitivity, costly fluorescence amplification and a weak fluorescence signal. Therefore, it is necessary to develop a highly sensitive and specific aptamer-based method with improved signal amplification for CRP detection.

Aptamers are single-stranded nucleic acids isolated from random-sequence DNA or RNA libraries by an *in vitro* SELEX,^19^ which can bind to various kinds of targets, including ions, proteins, whole cells and small molecules, with high specificity, sensitivity and affinity.^20^ They have attracted lot of interest because of their several advantages similar to antibodies,^10^ as well as their relative ease of isolation, modification, tailored binding affinity, chemical synthesis and resistance against denaturation.^3^ Aptamers belong to the group of so-called “functional nucleic acids” possessing catalytic or receptor properties.^9^ Short single-stranded DNA or RNA oligonucleotides can specifically bind to their targets, which favor them for protein detection, drug delivery and as new biorecognition elements for biosensing of disease-related proteins.^3^ Aptamers have displayed several advantages over antibodies in previous studies: they are highly chemically stable, show high detection sensitivity and selectivity and can be selected *in vitro* for nearly any given target.^21^ For these reasons, they are widely used as an alternative to antibody-based methods.

At present, graphene oxide (GO), a one-atom-thick^22^ 2D carbon nanomaterial^23^ with a honeycomb structure,^22^ has been used widely for the fluorescent biosensing strategy because of its excellent physical, chemical, thermal and mechanical properties.^24^ GO, having better aqueous solubility, is also under huge investigations as a potential materials in biomedicine.^25^ Due to the large surface area^23^ and biocompatibility,^26^ which make it possible to be loaded with many molecules *via* physical adsorption or chemical coupling, such as nucleic acids, proteins, and pharmaceutical molecules.^27^ The interaction between nucleic acid and GO includes two aspects, it has been proven that GO binds to DNA through π–π stacking^28^ interaction between the ring structures in nucleobase and the hexagonal cells of GO, and electrostatic repulsion occurs between phosphoric acid group of nucleic acid and carboxyl group of GO, so it can strongly bind single-stranded nucleic acids^29^ but rarely adsorbs double-stranded nucleic acids or oligonucleotides.^30^ Moreover, GO is a new nanomaterial with superior fluorescence quenching capacity,^31^ the fluorescence of the fluorophore labels of the surface adsorbed oligonucleotides can be quenched almost completely resulting in a fluorescently silent GO and single stranded oligonucleotide nanoassembly.^32^ Recently, a detailed investigation on the affinity difference of GO for ssDNA containing different numbers of bases in the length proved that short ssDNA had weaker affinity to GO than long ssDNA.^33^ Hence, GO, as a superb nanomaterial, has great potential as a highly sensitive substrate in biosensors^20^ for biochemical analysis.^24^

In our experiment, ribonuclease H (RNase H) also plays an important role. RNase H is a ribonuclease,^34^ which cleaves RNA phosphodiester bonds only when they present in an RNA–DNA heteroduplex; it does not digest the DNA in the heteroduplex, nor does it digest single- or double-stranded RNA or DNA.^35^ Members of the RNase H family can be found virtually in all organisms, from archaea and prokaryotes to eukaryotes, as well as in many important biological processes, such as DNA replication, DNA repair, and transcription.^36^ RNase H is also tightly associated with the retroviral reverse transcription process and drug resistance.^37^ The specific catalytic functions of RNase H are involved in a number of important biological processes including DNA replication, DNA repair, and transcription.^38^ More importantly, it can be used as a tool enzyme in molecular biology and biotechnology, *e.g.* DNA detection, removal of mRNA from an mRNA: cDNA duplex, RNA isotope labeling and specific cleavage of RNA in synthetic DNA.^39^ In addition, there have been some reports in recent years that develop an RNase H-assisted signal amplification for the rapid and reliable detection of biomolecule.^40^ Therefore, RNase H-aided signal amplification allows improved sensitivity and availability. The development of an RNase H-aided signal amplification strategy has great potential for application in aptamer-based CRP detection with improved specificity and sensitivity.

In this study, we combine the specificity offered by aptamer-based biosensors and the RNase H recycling single amplification to design a specific and sensitive biosensor. We develop an innovative aptamer-based method for the ultrasensitive fluorescence detection of CRP using the RNase H assisted DNA recycling amplification strategy. In this assay, in the presence of CRP, it can specifically bind to aptamer of CRP and then the DNA chain of P1 release from the Ap/P1 complexes. With the addition of the fluorescence labeled (5-FAM) RNA, P1 hybridizes with fluorescence labeled RNA to form P1/RNA double strand. When the RNase H was added, the RNA with fluorescent labeling in the double strand was specifically cut into nucleotide fragments, which cannot adsorb on the surface of the GO, so as to generate fluorescence. As a result, the reaction repeats cycles until the RNase H is consumed, and thus amplifying the fluorescence signal. Furthermore, this simple and fast amplification strategy is fast without complex steps, and provides an innovative method for detection of CRP. Our biosensor offers a rapid, sensitive and specific way^41^ to detect CRP, which has great potential for the diagnosis, prognosis and treatment of the disease.

## 2 Experimental section

### 2.1 Reagents and materials

The aptamer (5′-GGCAGGAAGA CAAAC ATATAATT GAGATCG TTTGATGAC TTTGTAAGAGTGTGGAATGGTCTGTGGTGCTGT-3′), DNA chain of P1 (5′-AGCACCACAGACCATTCCACACT-3′), 5-FAM labeled RNA (5′-AGUGUGGAAUGGUCUGUGGUGCU-3′) was purchased from the Takara (Dalian, China), the RNase H was obtained from the Thermo Scientific and GO was purchased from XFNANO Co. Ltd. (Nanjing, China, https://www.xfnano.com). All of the reagents were diluted to the required concentration with working buffer (20 mM Tris–HCl, 40 mM KCl, 8 mM MgCl_2_, 1 mM DTT, pH 7.8) before use. The other reagents were of analytical grade and used without further purification. This study all experiments were performed in strict accordance with the relevant laws and institutional guidelines (National Health Commission of the people's Republic of China, ethical review of biomedical research involving human beings). The biological samples such as healthy human serum, urine and saliva were obtained from Affiliated Dongfeng General Hospital, Hubei University of Medicine, and approved by Dongfeng General Hospital's Ethics Committee (Shiyan, China), informed consent was obtained for any experimentation with human subjects. Ultrapure water obtained from a Millipore water purification system (18.2 MΩ cm resistivity, Milli-Q Direct 8) was used in all runs.

### 2.2 Procedures for CRP detection

Firstly, the aptamer (Ap) of CRP and DNA (P1) were added and mixed in equal quantity, the Ap and the P1 were partial complementarity to form the Ap/P1 complexes. Secondly, the Ap/P1 were incubated with CRP for 20 min, the CRP and the Ap specifically bind to each other in this process. After incubation, the fluorescent labeled RNA were added and hybridized with P1 to form RNA/P1 complexes. Then, after the addition of the RNase H, the fluorescent labeled RNA to be cut by RNase H in the RNA/P1 double strand. Finally, added GO in the end, which has fluorescence quenching ability and can strongly adsorb single-stranded nucleic acids. So, P1 adsorbed onto the surface of GO *via* π–π stacking, but the short fragments of fluorescent labeled RNA cannot adsorb to the GO. Lastly, detect the fluorescence intensity of the mixed solution.

### 2.3 Fluorescence measurements

The fluorescence detection of the compounds was carried through a Hitachi F-4600 spectrophotometer (Hitachi Co. Ltd., Japan, https://www.hitachi.co.jp) equipped with a xenon lamp excitation source at room temperature. The excitation spectra were set at 495 nm and the emission spectra were collected from 480 nm to 600 nm. The fluorescence intensity at 519 nm was used to studied the optimal experimental parameters and evaluate the function of the designed sensing system. The intervals of the excitation and emission were both set at 5 nm. Excepted for special groups, the fluorescence measurements were repeated three times for each group and calculated the standard deviation. The quantitative determination of CRP was expressed by fluorescence intensity. *F*_1_ and *F*_0_ are the fluorescence intensities at 519 nm in the presence and absence of CRP, respectively.

## 3 Results and discussion

### 3.1 Detection mechanism

**Fig. 1:**
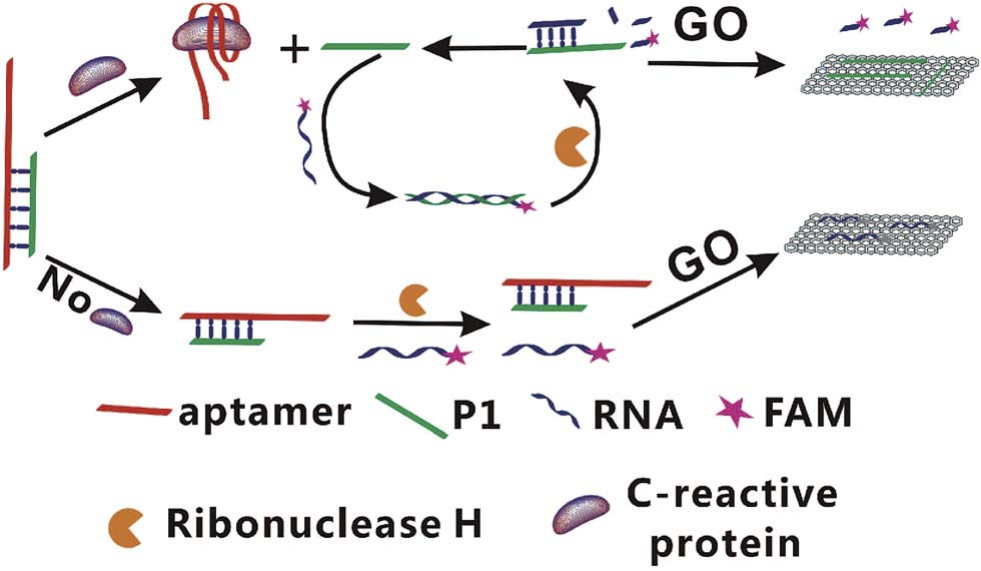
Schematic illustration of the GO-based fluorescent aptasensor assay for the detection of CRP based on RNase H target recycling signal amplification (GO: graphene oxide; P1: DNA chain; FAM: 5-carboxy-fluorescein).

In present study, a fluorescence amplification method of sensitive CRP detection combined with aptamer-based CRP recognition strategy was developed. As shown in Fig. 1, strand P1, which can be nonspecifically bind with the partial sequence of aptamer to form the Ap/P1 complexes. In the presence of CRP, the aptamer is released to form the Ap/P1 complexes and then specifically binds with CRP to form the CRP/Ap complexes. In this process, P1 also dissociates from the Ap/P1 complexes. After the addition of the fluorescence labeled RNA, P1 can hybridize with the fluorescent labeled RNA to form P1/RNA double strand. The RNase H, which has ability to cut the RNA chain specifically in the DNA/RNA chains, but does not cut RNA itself and the phosphodiester bond in the single strand or double strand DNA. Therefore, after the addition of RNase H, the fluorescent labeled RNA in P1/RNA double strand was cut into short fragments of nucleotides, and P1 in the P1/RNA double strand was released and repeat this cycle. At the same time, GO, which has fluorescence quenching ability and can strongly adsorb single-stranded nucleic acids. It does not bind with double-stranded nucleic acid, and the affinity of long-chain DNA to GO is better than that of short chains. Thus, P1 adsorbed onto the surface of GO *via* π-stacking. The short fragments of fluorescent labeled RNA cannot adsorb to the GO, and thus produce the fluorescent signal. Therefore, the strong fluorescence intensity verifies the existence of CRP.

On the contrary, in the absence of CRP, the Ap/P1 complexes cannot be cut by RNase H, the fluorescent labeled RNA cannot hybridize with P1 to form double strands and thus it can be adsorbed by GO, resulting in a quenched fluorescence.

### 3.2 Feasibility analysis of the developed method for CRP detection

**Fig. 2:**
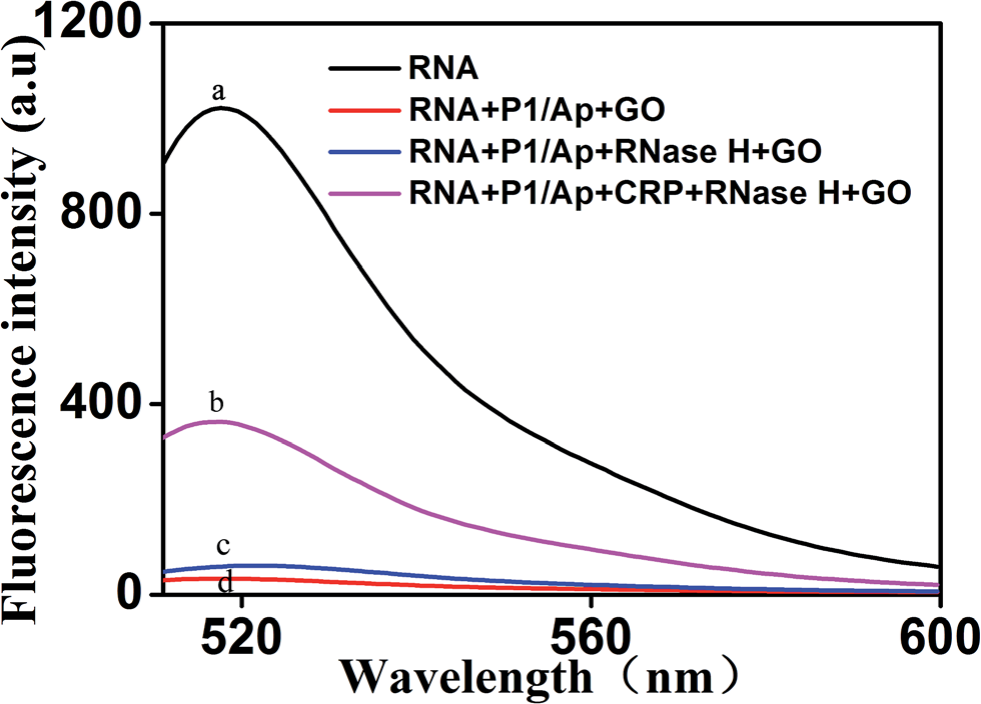
Representative fluorescent emission spectra of different mixtures. From curve a to d: (a) RNA; (b) RNA + P1/Ap + CRP + RNase H + GO; (c) RNA + P1/Ap + RNase H + GO; (d) RNA + P1/Ap + GO. The concentrations of RNA, P1 and GO were 50 nM, 20 nM and 20 μg mL ^−1^, respectively.

To further verify the feasibility of the fluorescence signal amplification strategy, fluorescence measurements were performed to record the fluorescence emission spectra of different mixtures. As shown in Fig. 2, the fluorescence intensity of fluorescent labeled RNA (curve a) is relatively strong. After adding the Ap/P1 (curve d) or RNase H (curve c), the fluorescence intensity was relatively weak in the presence of GO. Because the fluorescent labeled RNA does not react with Ap/P1 or RNase H under this condition, fluorescent labeled RNA adsorbed on the surface of GO and the fluorescence was quenched by GO. After adding the CRP in the mixture solution of RNA, RNase H and P1/Ap in the presence of GO (curve b), the CRP react with Ap to form the CRP/Ap complexes. And the RNase H cut the RNA chain specifically in the P1/RNA chains to form the short fragments of nucleotides, which cannot adsorb on the surface of GO and thus produce the fluorescent signal. These results confirm the feasibility of the proposed fluorescence biosensor for CRP detection by this design.

**Fig. 3:**
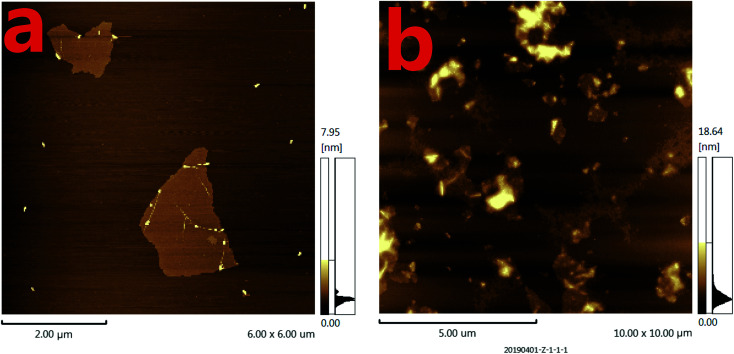
The atomic force microscopy (AFM) images of different mixtures. (a) The AFM image of GO dripping directly on the surface of mica; (b) the AFM image of the mixtures of GO with microRNAs on the surface of mica. The concentrations of GO and microRNAs were 20 μg mL^−1^ and 20 nM, respectively. The volume of GO and microRNAs were both of 6 μL.

On the other hand, the morphology of the formed GO as well as the mixture of GO and miRNA on the mica were characterized by atomic force microscopy (AFM). Tapping mode AFM images were acquired under ambient conditions by using a Shimadzu SPM9700 AFM system. Fig. 3(a) shows the results of GO dropping directly on the surface of mica and then drying. Under the same conditions, as shown in Fig. 3(b), GO was dripped directly on the surface of mica and dried. Then, the microRNAs with a concentration of 20 nM were added to the surface of mica and placed for 10 minutes. Finally, wash the mica with water and dry it. It can be seen from the Fig. 3(b) that a large amount of microRNAs are adsorbed on the surface of GO. Therefore, the above results show that GO has a strong adsorption capacity for single-stranded nucleotides, which also proves the feasibility of the proposed biosensor.

### 3.3 Optimization of experimental conditions

In order to achieve the high sensitivity performance for CRP detection, several reaction conditions in this method such as the concentration of RNA, the concentration of P1, the amount of RNase H, and the enzyme digestion time were optimized. The fluorescence intensity and the value of *F*_1_/*F*_0_ were selected to evaluate the effects of the aforementioned reaction conditions on the sensing performance of the developed strategy, where *F*_1_ and *F*_0_ were the fluorescence intensities of the solutions in the presence and absence of CRP, respectively.

**Fig. 4:**
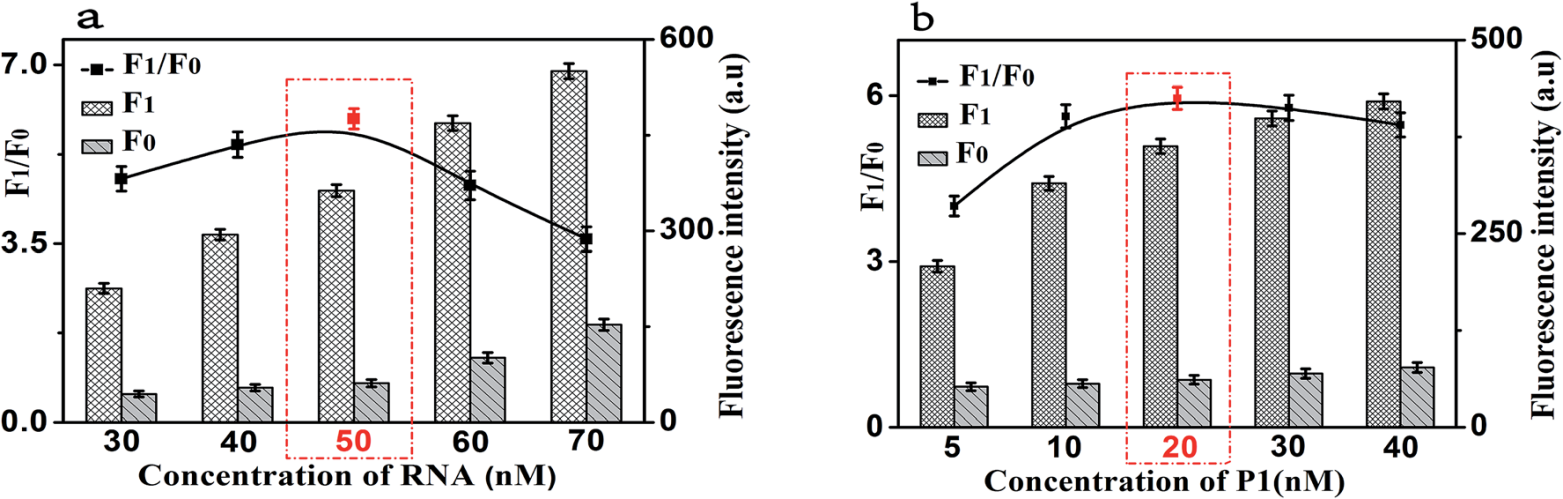
(a) The fluorescence response as a function of RNA concentration. (b) The fluorescence response as a function of P1 concentration. *F*_0_: control experiments; *F*_1_: with 10 ng mL^−1^ of CRP. The black lines represent the *F*_1_/*F*_0_ at different conditions, while the *F*_1_ and *F*_0_ were the fluorescence intensities in the presence and absence of CRP, respectively. Error bars: SD, *n* = 3.

Firstly, the concentration of RNA plays an important role in the performance of this sensing method. The results shown in Fig. 4(a) displayed the changes in fluorescence intensity with different concentrations of RNA. The maximum *F*_1_/*F*_0_ value was observed when the concentration of GO was 50 nM. When the concentration of RNA was further increased, however, the *F*_1_/*F*_0_ value significantly decreased with the increasing RNA concentration. Therefore, the concentration of 50 nM was selected as the optimized RNA concentration. Next, the concentration of P1 also affects the fluorescence intensity of the mixed solution. Thus, we optimized the concentration of P1 and the results are shown in Fig. 4(b). With the increase of the concentration of P1, the fluorescence intensity increased gradually and then decreased. The maximum *F*_1_/*F*_0_ value was observed when the concentration of P1 is 20 nM. Therefore, the optimal concentration of P1 is 20 nM, which can be used for the following experiments.

**Fig. 5:**
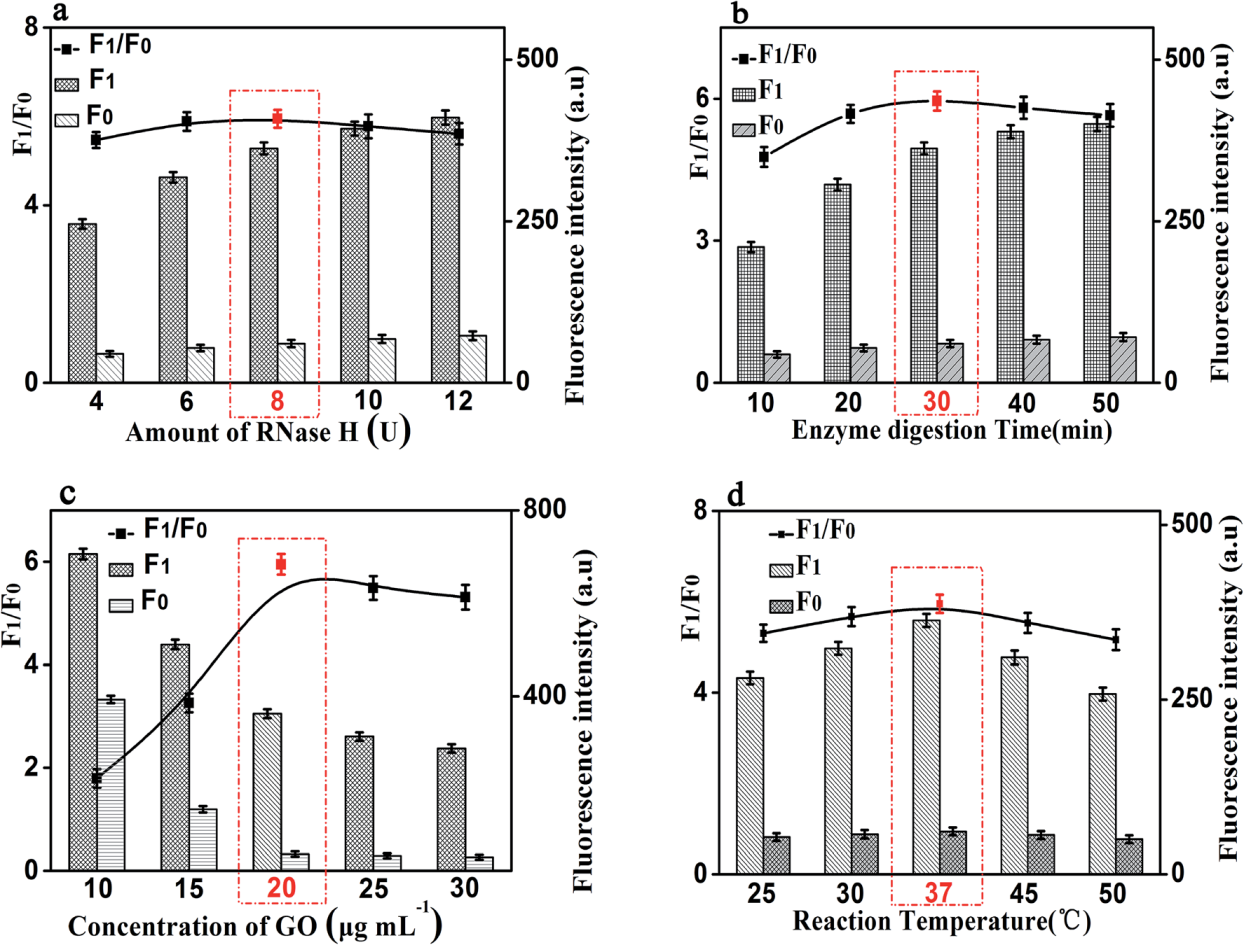
Effects of reaction conditions on the fluorescent signals and *F*_1_/*F*_0_ values of the proposed method. (a) Amount of RNase H (U). (b) Enzyme digestion time (min). (c) Concentration of GO (μg mL^−1^). (d) Reaction temperature (°C). *F*_0_: control experiments; *F*_1_: with 10 ng mL^−1^ of CRP. The black lines represent the *F*_1_/*F*_0_ at different conditions, while the *F*_1_ and *F*_0_ were the fluorescence intensities in the presence and absence of CRP, respectively. Error bars: SD, *n* = 3.

It is worth noting that the amount of RNase H also affects the fluorescence intensity of this experiment. We studied the fluorescence intensity at different amount of RNase H and Fig. 5(a) showed that the fluorescence signal was maximum when the amount of RNase H is 8 U. So, we chose the amount of RNase H with 8 U as the optimized amount. Finally, the enzyme digestion time is another significant reaction parameter affecting the fluorescence intensity for our experiment. As shown in Fig. 5(b), with the increase of the enzyme digestion time, the fluorescence intensity increased first and then decreased. The maximum *F*_1_/*F*_0_ values was observed when the enzyme digestion time was 30 min. Thus, 30 min of the enzyme digestion time was selected for the rest of our experiments.

Besides the above conditions, the concentration of GO and the reaction temperature may also affect the fluorescence intensity. As shown in Fig. 5(c), it shows that the fluorescence intensity increased first and then decreased with the increase of the concentration of GO, and the *F*_1_/*F*_0_ value reached to the maximum when the concentration of GO is 20 μg mL^−1^. Therefore, 20 μg mL^−1^ was be treated as the optimized reaction concentration of GO. In addition, Fig. 5(d) shows that the temperature of the reaction also affects the fluorescence intensity. The fluorescence intensity increased first and then decreased with the increasing of temperature, and when the temperature reached 37 °C, the *F*_1_/*F*_0_ value reached its maximum value. Therefore, the reaction temperature of 37 °C was selected in the experimental design.

### 3.4 Sensitivity for CRP detection

**Fig. 6:**
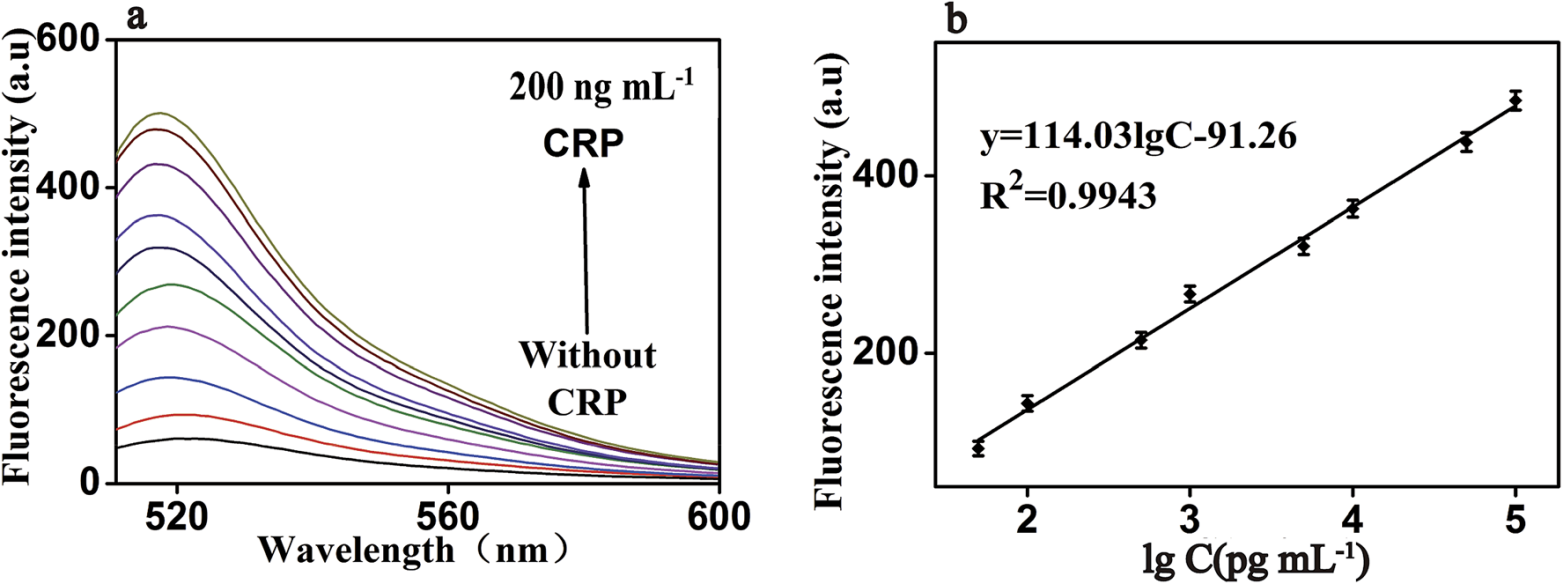
(a) Fluorescence emission spectra of the biosensor in the presence of CRP with different concentrations: from bottom to top: 0 to 200 ng mL^−1^ (b) The fluorescence intensity as a function of CRP concentration. It shows the strong correlation between fluorescence intensity and CRP concentration, under both experimental conditions: RNA, 50 nM; P1, 20 nM; RNase H, 8 U; GO, 20 μg mL^−1^ and the emission wavelength of 519 nm. Error bars: SD, *n* = 3.

Under the optimal reaction conditions, the sensitivity of the proposed fluorescence intensity amplification strategy for CRP detection was evaluated at different concentrations. As shown in Fig. 6(a), we found that the fluorescence intensity increased with the increasing concentration of CRP, from 0 to 200 ng mL^−1^. It shows the concentration of CRP has a strong correlation with the fluorescence intensity. At the same time, Fig. 6(b) shows that a good linear correlation between the fluorescence intensity and the logarithm of CRP concentration was observed, in the range from 50 pg mL^−1^ to 100 ng mL^−1^. The regression equation was expressed as *y* = 114.03 log *c* − 91.26 (*R*^2^ = 0.9943), where log *c* is the logarithm of CRP concentration (pg mL^−1^). The detection limit of CRP was calculated to be 0.01 ng mL^−1^ (according to the 3S rule). As presented in Table 1, CRP detection performance of our strategy was comparable to the reported in most of the other studies.^3–18^

**Table 1:** The comparison between our method and other reported biosensors for the detection of CRP

Analytical method	Detection limit	Linear range	Ref.
Electrochemical	54 ng mL^−1^	0.1–50 μg mL^−1^	7
Electrochemical	1.5 ng mL^−1^	0.01–150 μg mL^−1^	8
Electrochemical	0.115 ng mL^−1^	115 pg mL^−1^ to 11.5 ng mL^−1^	9
Electrochemical impedance spectroscopy	3.4 × 10^−7^ ng mL^−1^	21 fg mL^−1^ to 6.148 pg mL^−1^	18
Microfluidic biosensor	12.5 ng mL^−1^	12.5 ng mL^−1^ to 10 μg mL^−1^	6
Microfluidic biosensor	340 ng mL^−1^	0.625–10 μg mL^−1^	5
Microfluidic biosensor	12.5 ng mL^−1^	12.5 ng mL^−1^ to 10 μg mL^−1^	4
Sandwich immunoassay	1 ng mL^−1^	0.01–100 μg mL^−1^	10
Sandwich immunoassay	400 ng mL^−1^	0.4–10 μg mL^−1^	11
Nanowire-mesh biosensor	9.03 × 10^−8^ ng mL^−1^	1.15 ag mL^−1^ to 115 μg mL^−1^	17
Magnetoimpedance-based biosensor	1.0 × 10^−3^ ng mL^−1^	1 pg mL^−1^ to 100 ng mL^−1^	16
Capacitive biosensor	115 ng mL^−1^	0.115–1.15 μg mL^−1^	3
Fiber optic biosensor	62.5 ng mL^−1^	62.5 ng mL^−1^ to 1 μg mL^−1^	15
Chemiluminescence immunoassay	130 ng mL^−1^	0.3–160 μg mL^−1^	13
Micro-fluxgate biosensor	2 ng mL^−1^	2 ng mL^−1^ to 10 μg mL^−1^	14
Fluorescent biosensor	43.7 ng mL^−1^	57.5 ng mL^−1^ to 2.3 μg mL^−1^	12
Fluorescent biosensor	0.01 ng mL^−1^	50 pg mL^−1^ to 100 ng mL^−1^	This method

### 3.5 Specificity for CRP detection

**Fig. 7:**
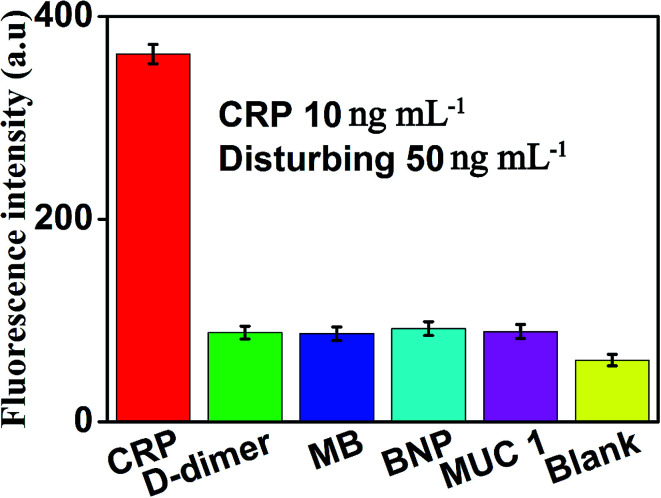
Fluorescence intensity (at the emission wavelength of 519 nm) of the sensor in the presence of CRP (10 ng mL −1), D-dimer (50 ng mL^−1^), MB (50 ng mL^−1^), BNP (50 ng mL^−1^), MUC 1 (50 ng mL^−1^) and blank, respectively. Error bars: SD, *n* = 3.

The specificity of this method for CRP detection was also studied by adding five different types of proteins including CRP (10 ng mL^−1^), D-dimer (50 ng mL^−1^), MB (50 ng mL^−1^), BNP (50 ng mL^−1^) and MUC 1 (50 ng mL^−1^). Measuring these five different proteins under the same conditions to validate the specificity of our method for CRP detection. The results of the specific study are shown in Fig. 7, compared with the blank control group, negligible fluorescence signal was observed in the presence of D-dimer, MB, BNP or MUC 1. However, the fluorescence signal increased significantly after the addition of 10 ng mL^−1^ CRP, which indicated that this proposed strategy showed the satisfactory specificity for CRP detection. The excellent specificity of the aptamer-based biosensor for CRP detection is mainly ascribed to the specific recognition and high affinity between the aptamers and the targets.

### 3.6 Determination of CRP in real samples

**Fig. 8:**
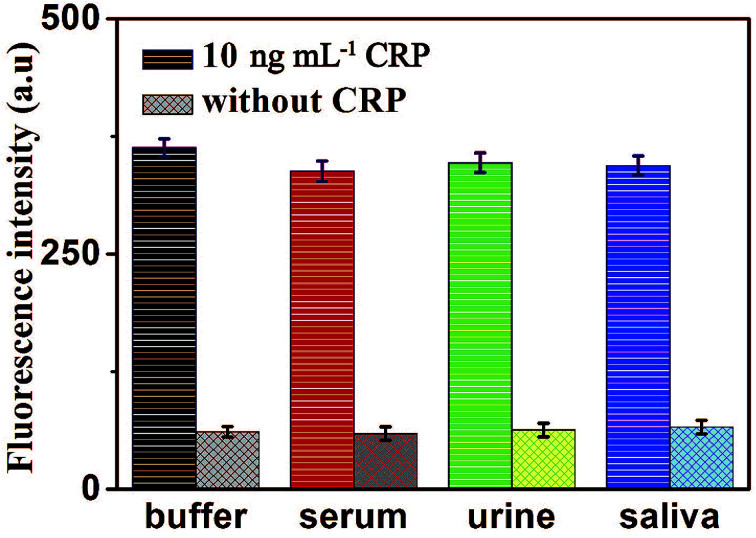
Fluorescence intensity of the sensor for the detection of CRP in buffer and blank biological samples (human serum, urine and saliva, respectively). Error bars: SD, *n* = 3.

To further verify the potential applicability and feasibility of the present strategy in biological samples, human, serum, urine and saliva, respectively, were employed as a complex matrix. The detection of CRP in biological samples by spiking in CRP to human serum, urine and saliva diluted to 10% with buffer solution with the final concentration of 10 ng mL^−1^ were performed. Moreover, a group of blank biological samples without adding CRP was set up to detect its fluorescence intensity. As shown in Fig. 8, a significantly enhancement in fluorescence intensity of various biological samples was observed in the presence of 10 ng mL^−1^ CRP, compared with the negligible fluorescence intensity of the blank biological samples. These results indicated that the detection of CRP in biological samples is free of matrix interference, and this biosensor can be a potential and feasible analytical method to detect CRP in the real biological samples sensitively.

## 4 Conclusion

In summary, a simple, specific and highly sensitive strategy of fluorescent signal amplification was successfully developed for the detection of CRP. In this design, due to a significant fluorescent amplification signal in response to CRP and aptamer-based CRP recognition, the highly sensitive and specific detection of CRP was achieved. This sensor can detect CRP sensitively with a linear range from 50 pg mL^−1^ to 100 ng mL^−1^, and the detection limit was as low as 0.01 ng mL^−1^. It is worth noting that our method has been successfully applied in the specific and sensitive detection of CRP in all kinds of real biological samples, indicating that it has the potential to become a reliable approach for CRP detection in the early clinical diagnosis of disease. Furthermore, the present ultrasensitive fluorescence amplification biosensor strategy may be also a potential strategy for the direct detection of other biomarkers by selecting the appropriate aptamers in the early clinical diagnosis.

## Conflicts of interest

There are no conflicts to declare.

## Supplementary Material
