# Correction: Ultrasensitive fluorescent aptasensor for CRP detection based on the RNase H assisted DNA recycling signal amplification strategy

**DOI:** 10.1039/d2ra90046g

**Published:** 2022-04-27

**Authors:** Zhongzhi Liu, Dan Luo, Fangling Ren, Fengying Ran, Wei Chen, Bingqiang Zhang, Ceming Wang, Hao Chen, Jian Wei, Qinhua Chen

**Affiliations:** Affiliated Dongfeng Hospital, Hubei University of Medicine Hubei Shiyan 442008 China cqh77@163.com +86 0719 8272283; College of Pharmacy, Hubei University of Medicine Hubei Shiyan 442008 China; Hubei Key Laboratory of Wudang Local Chinese Medicine Research, Hubei University of Medicine Hubei Shiyan 442008 China

## Abstract

Correction for ‘Ultrasensitive fluorescent aptasensor for CRP detection based on the RNase H assisted DNA recycling signal amplification strategy’ by Zhongzhi Liu *et al.*, *RSC Adv.*, 2019, **9**, 11960–11967, https://doi.org/10.1039/C9RA01352K.

The authors are publishing this correction to draw the reader’s attention to their closely related papers that should have been cited as ref. 42 and 43 in this *RSC Advances* article. These are given below as ref. 1 and 2, respectively.

On page 11963, a citation to ref. 42 should be added to the end of the sentence beginning “The fluorescence intensity…”. Therefore, the sentence should be changed to “The fluorescence intensity and the value of *F*_1_/*F*_0_ were selected to evaluate the effects of the aforementioned reaction conditions on the sensing performance of the developed strategy, where *F*_1_ and *F*_0_ were the fluorescence intensities of the solutions in the presence and absence of CRP, respectively.^42^”

On page 11965–11966, a citation to ref. 43 should be added at the end of the sentence beginning “The detection of CRP…”. Therefore, the sentence should be changed to “The detection of CRP in biological samples by spiking in CRP to human serum, urine and saliva diluted to 10% with buffer solution with the final concentration of 10 ng mL^−1^ were performed.^43^”

The authors also regret that there are portions of unattributed text overlap in the Introduction, Results and discussion and Conclusion sections with other papers, including ref. 42 and 43.

The Royal Society of Chemistry apologises for these errors and any consequent inconvenience to authors and readers.

## Supplementary Material
